# Clusters of competence: Relationship between self‐reported professional competence and achievement on a national examination among graduating nursing students

**DOI:** 10.1111/jan.14222

**Published:** 2019-10-28

**Authors:** Henrietta Forsman, Inger Jansson, Janeth Leksell, Margret Lepp, Christina Sundin Andersson, Maria Engström, Jan Nilsson

**Affiliations:** ^1^ School of Education, Health and Social Studies Dalarna University Falun Sweden; ^2^ Institute of Health and Care Sciences Sahlgrenska Academy University of Gothenburg Gothenburg Sweden; ^3^ Department of Medical Sciences, Clinical Diabetology and Metabolism Uppsala University Uppsala Sweden; ^4^ Østfold University College Halden Norway; ^5^ School of Nursing and Midwifery Griffith University Brisbane Australia; ^6^ Department of Health Science, Nursing Faculty of Health, Science and Technology Karlstad University Karlstad Sweden; ^7^ Faculty of Health and Occupational Studies University of Gävle Gävle Sweden; ^8^ Nursing Department Medicine and Health College Lishui University Lishui China; ^9^ Department of Public Health and Caring Sciences Uppsala University Uppsala Sweden; ^10^ Department of Health Promotion Sciences Sophiahemmet University Stockholm Sweden; ^11^ Japanese Red Cross Institute for Humanitarian Studies Tokyo Japan

**Keywords:** cluster analysis, nursing education, nursing students, professional competence, questionnaires, self‐assessment, survey

## Abstract

**Aims:**

To identify clusters based on graduating nursing students’ self‐reported professional competence and their achievement on a national examination. Furthermore, to describe and compare the identified clusters regarding sample characteristics, students’ perceptions of overall quality of the nursing programme, and students’ general self‐efficacy (GSE).

**Design:**

A cross‐sectional study combining survey data and results from a national examination.

**Methods:**

Data were collected at two universities and one university college in Sweden in January 2017, including 179 students in the final term of the nursing programme. The study was based on the Nurse Professional Competence Scale, the GSE scale, and results from the National Clinical Final Examination. A two‐step cluster analysis was used to identify competence profiles, followed by comparative analyses between clusters.

**Results:**

Three clusters were identified illustrating students’ different competence profiles. Students in Clusters 1 and 2 passed the examination, but differed in their self‐assessments of competence, rating themselves under and above the overall median value, respectively. Students in Cluster 3 failed the examination but rated themselves at the overall median level or higher.

**Conclusion:**

The study illustrates how nursing students’ self‐assessed competence might differ from competency assessed by examination, which is challenging for nursing education. Self‐evaluation is a key learning outcome and is, in the long run, essential to patient safety.

**Impact:**

The study has identified clusters of students where some overestimate and others underestimate their competence. Students who assessed their competence low but passed the exam assessed their GSE lower than other students. The findings illuminate the need for student‐centred strategies in nursing education, including elements of self‐assessment in relation to examination to make the students more aware of their clinical competence.

## Abstract (zh)

**目的:**

此研究旨在根据应届护理专业学生自我报告的专业能力及其在全国考试中的成绩来确定类别。此外还需对样本特征、学生对护理专业课程总体质量的看法以及学生的一般自我效能感(GSE)的已确定特征进行描述和比较。

**设计:**

一项结合了调查数据和国家考试结果的横断面研究。

**方法:**

2017年1月,我们收集了瑞典的两所大学和一所大学学院的数据,其中包括179名护理专业课程的应届毕业生。此项研究基于护士专业能力量表、一般自我效能感(GSE)量表以及国家临床期末考试。我们使用两步聚类分析法以确定能力性质,随后进行类别之间的比较分析。

**结果:**

此项研究最终确定了三个类别,分别说明学生的不同能力性质。类别1和类别2的学生通过了考试,但他们的自我能力评估有所不同,他们分别将自己评定为总体中位值以下和以上。类别3的学生未能通过考试,但他们认为自己处于总体中位水平或更高。

**结论:**

此项研究探讨了护理学生的自我能力评估可能与考试评定的能力可能存在怎样的差异,这对护理教育来说是一个挑战。自我评估是关键的学习成果,并且从长远来看,对患者安全至关重要。

**影响:**

此项研究确定了学生类别,其中部分学生高估了自己的能力,部分学生低估了自己的能力。自我评估能力较低但通过考试的学生所评定的一般自我效能感(GSE)要比其他学生低。研究结果表明需要在护理教育中使用以学生为中心的策略,包括与考试相关的自我评估要素,从而使得学生更清楚认识到自己的临床能力。

## 1 INTRODUCTION

To contribute to a global future workforce of nurses with professional competence in providing safe and high‐quality care, it is important to measure and evaluate nursing students’ progress and achievements towards educational goals and requirements. Structured methods including different evaluation tools are common in evaluations of nursing students’ clinical competence (Lejonqvist, Eriksson, & Meretoja, 2016). However, validity and reliability in assessments of competence may vary and pre‐existing expectations from mentors, in addition to their shared understanding of educational goals, are present in this assessment process (Burden, Topping, & O´Halloran, 2018). The ability to identify one's own knowledge gaps and need for competence development is a competence that is clearly formulated in the educational goals for Swedish nursing programmes (Higher Education Ordinance (1993:100). Self‐evaluations can be operationalized in terms of, for example, perceived knowledge or performance/skill evaluation and the overall correspondence between self‐evaluated ability and objective performance outcomes is moderate (Zell & Krizan, 2014). Previous studies have shown how assessments of students’ achievements might differ between students’ self‐assessments on the one hand and assessments by preceptors (Ugland Vae, Engström, Mårtensson, & Löfmark, 2018), examiners (Baxter & Norman, 2011), and structured clinical examinations (Sears et al., 2014) on the other hand.

### 1.1 Background

Quality and safety competencies for nurses have been defined by the Quality and Safety Education for Nurses (QSEN) and the National Advisory Board (Cronenwett et al., 2007) and are based on core professional competencies described by the Institute of Medicine (2003). QSEN has proposed targets for knowledge, skills, and attitudes to be developed for each of the following competencies: patient‐centered care, teamwork and collaboration, evidence‐based practice, quality improvement, safety, and informatics. These six competencies can serve as guidelines in the curricular development of formal academic programmes, transition to practice, and continued study programmes (Cronenwett et al., 2007). The Swedish Society of Nursing (2017) has adopted these six core competencies, along with leadership and education, in a description of competence for Swedish nurses. Research shows the association between nurses’ competence and patient outcomes (Kendall‐Gallagher, Aiken, Sloane, & Cimiotti, 2011) and that academic competence (Aiken et al., 2014) and a higher proportion of professional nurses (Aiken et al., 2017) are associated with better outcomes for patients. A recently published report by The Swedish National Board of Health and Welfare (2018) highlights how a lack of competence among healthcare staff significantly increases the risk of patient injuries, due to patients being exposed to danger. However, the concept of competence lacks a clear, coherent, and comprehensive definition or description (Kajander‐Unkuri, Salminen, Saarikoski, Suhonen, & Leino‐Kilpi, 2013; Liu & Aungsuroch, 2018; Nehrir, Vanaki, Mokthari Nouri, Khademolhosseini, & Ebadi, 2016).

Nursing programmes in Sweden consist of 3 years of full‐time studies (180 ECTS credits) with both theoretical and clinical studies included. Nursing programmes are offered at 25 Higher Education Institutions (HEI) and graduating students are awarded with a professional nursing degree and a Bachelor of Science degree. The objectives for Nursing Education are stipulated in the Swedish Higher Education Act (1992:1434) and the Swedish Higher Education Ordinance (1993:100) and are operationalized by each respective HEI.

The Miller's pyramid for clinical competence (Miller, 1990) provides a framework for assessing students’ depth of knowledge and performance at four levels; *Knows* refers to the student having sufficient knowledge of the field they will be working in and *Knows how* is when the student can apply this knowledge. These steps can be tested with a written examination. *Shows how* is when the student can demonstrate how a procedure is performed in an artificial environment. *Does* is when students can perform tasks professionally in an authentic context. Levels 3 and 4 can be tested through clinical exams and direct observation in clinical settings.

In Sweden, work on introducing a voluntary national clinical examination started in 2003 (Athlin, Larsson, & Söderhamn, 2012) and now, 14 of the 25 HEIs with nursing programmes conduct The National Clinical Final Examination (NCFE). The aim of the NCFE is to examine third‐year nursing students’ clinical competence to ensure that they have the clinical knowledge and skills required, as laid out in the national legislation, before they graduate the programme and enter working life. The NCFE has a written and a bedside test and is structured so all the steps in Miller's pyramid (Miller, 1990) can be reached. Evaluation of the NCFE shows that the model measures the level of competence of nursing students and that its design is beneficial for the students’ clinical reasoning (Ziegert, Elmqvist, Johansson, Larsson, & Andersson, 2014).

While the NCFE represents competence assessed by examination, self‐assessed competence is measured using the Nurse Professional Competence Scale (NPC) (Gardulf et al., 2019, 2016; Nilsson et al., 2016, 2014; Theander et al., 2016). This scale has recently been developed into the NPC short form (NPC‐SF) that covers six competence areas: Nursing Care, Value‐based Nursing Care, Medical and Technical Care, Care Pedagogics, Documentation and Administration of Nursing Care and Development, Leadership and Organization of Nursing Care (Nilsson, Engström, Florin, Gardulf, & Carlsson, 2018).

Self‐evaluation of ability can also be operationalized in terms of self‐efficacy (Zell & Krizan, 2014). Self‐efficacy is the belief in one's capability to execute the behaviour required to produce desired outcomes. Self‐efficacy is a concept that includes belief in one's own ability to perform an action; the greater the level of self‐efficacy one has, the more likely it is that they will start and continue an activity with a positive result (Bandura, 1997).

Previous research illustrates the complex relationship between self‐perceived performance and actual performance and how the ability to make accurate self‐assessments might differ between students in nursing education (Burden et al., 2018; Lejonqvist et al., 2016; Ugland Vae et al., 2018). It is essential that this is studied further since correct self‐assessments are crucial to both learning and patient safety in clinical practice.

## 2 THE STUDY

### 2.1 Aims

The aim of the study was to identify clusters based on graduating nursing students’ self‐reported professional competence and their achievement on a national examination. An additional aim was to describe and compare the identified clusters regarding sample characteristics, students’ perceptions of overall quality of the BSN programme, and students’ general self‐efficacy (GSE).

### 2.2 Design

This was a cross‐sectional study, combining survey data and results from a national examination.

### 2.3 Sample/participants

All students taking the NFCE written and bedside exam and also responding to the questionnaires, in their final term of the nursing programme at two universities and one university college in Sweden were included in this study (*N* = 179).

### 2.4 Data collection

A research‐group representative at each HEI gave written and oral information about the study and issued questionnaires to nursing students during their last 2 weeks of the nursing programme (January, 2017). Students were given an envelope containing information about the study and a coded questionnaire, which was completed individually and then left in a designated box in the classroom. Using a code key, the research‐group representative at each HEI linked each questionnaire to a student's NFCE results.

To measure nursing students’ self‐rated competence, the NPC‐SF was used. Data were collected using a 35‐item version distributed in six competence areas: Nursing Care (five items, Cronbach's alpha (*α*) in the present study .79); Value‐Based Nursing Care (five items, *α* = .79); Medical and Technical Care (six items, *α* = .81); Care Pedagogics (five items, *α* = .87); Documentation and Administration of Nursing Care (eight items, *α* = .79) and Development, Leadership and Organization of Nursing Care (six items, *α* = .80). Response alternatives used a 4‐grade scale: 1 = to a very low degree, 2 = to a relatively low degree, 3 = to a relatively high degree, and 4 = to a very high degree. Scores for the respective competence areas were calculated by summing up all items divided by the highest possible score in the competence area and then multiplying by 100, thereby giving 0–100 values.

Self‐efficacy was measured using the Swedish version of the 10‐item GSE scale (Koskinen‐Hagman, Schwarzer, & Jerusalem, 1999; Schwarzer & Jerusalem, 1995). The 10 items are rated on a 4‐point Likert scale ranging from 1 = ‘not at all true’ to 4 = ‘exactly true’, where higher scores indicate a higher level of self‐efficacy.

Sociodemographic data included age, gender, previous education, and work while studying the BSN programme. The students were also asked about the overall quality of the programme and whether they would recommend the programme to others.

To measure the students’ competence by examination, results from the NCFE were used (Athlin et al., 2012). The written exam is a modified essay question exam, which means that as the examination proceeds, situations and conditions change and new questions are added (Khan & Aljarallah, 2011). To pass, students need 33 points of a possible 50 and two questions about drug calculation must be correctly answered. During the bedside examination, students take care of a patient in need of comprehensive medical and nursing care for 3 hr. An experienced Registered Nurse evaluates their performance based on a protocol following the nursing process. A clinical lecturer makes the final assessment that decides whether the student will pass or fail. Students must pass both the written and bedside tests to pass the examination. Students in this present study accomplished their written NCFE in November 2016 (same date and time for all HEIs) and their bedside examination during their final term of the nursing programme, that is, September 2016–January 2017.

### 2.5 Ethical considerations

The study was carried out in accordance with the World Medical Association declaration of Helsinki (originally adopted in 1964). A local ethics committee reviewed the study (Dnr C2016/567) and it was determined that the project did not fall under the Ethics Assessment Act (2003:460). Participants were informed via a letter of the aim of the study, that their participation was voluntary and that they were entitled to terminate their participation at any time. A response to the questionnaire was interpreted as consent from participants.

### 2.6 Data analysis

IBM SPSS Statistics version 22 was used for data analysis (SPSS Inc.). Data were screened for missing values and multiple imputation (MI) was used to handle internal missing data. The method generated five imputed datasets together with the original dataset. Students with more than 50% missing data in a factor of the NPC‐SF scale were excluded in all further analyses (*N* = 9). The final cluster sample resulted in 170 study participants, whereof 121 had complete responses/no missing data at all in NPC‐SF. Descriptive statistics were used to describe sample characteristics, results on the NPC‐SF and NCFE.

Cluster analysis was used to identify homogeneous clusters of participants based on their NPC‐SF competence profiles and achievement on the NCFE. The two‐step cluster analysis (TSCA) procedure was used with log‐likelihood distance measures. This method was chosen as it allows the use of both continuous and categorical variables and the method improves the weaknesses of applying a single clustering method. The advantage of the method is that it ‘integrates hierarchical and partitioning clustering algorithm with adding attributes to cluster objects’ (Shih, Jheng, & Lai, 2010, p.11). The method first pre‐clusters cases into small subclasses and then forms final clusters using hierarchical methods. The optimal number of clusters is determined automatically in the TSCA using Schwarz's Bayesian Information Criterion. The Silhouette measure of cohesion and separation is used to determine the quality of the cluster solution (Norusis, 2012). A silhouette value of less than 0.20 indicates a poor solution, 0.20–0.50 indicates a fair solution and over 0.50 indicates a good solution (Mooi & Sarstedt, 2011). Comparative analyses (one‐way ANOVA, Kruskal–Wallis test, Mann–Whitney *U* test and chi‐square test) were used to test statistical differences between clusters regarding sample characteristics and the variables recommending the BSN programme, overall quality of the BSN programme, and GSE. The significance level was set at *p* < .05.

### 2.7 Validity and reliability

Previous studies have shown that the NPC Scale can be used as a tool for quality assessments and improvements of nursing education programmes (Gardulf et al., 2019). Construct validity of NPC‐SF has been tested with principal component analysis and confirmative factor analysis where the factor solution explained 54% of the overall variance. Reliability measured as internal consistency showed *α* values >.70 for all competence areas (Nilsson et al., 2018). Also in the present study, the internal consistency of the NPC‐SF was high for all competence areas, with *α* values ranging between 0.79–0.87. The GSE scale has been validated in several languages and is widely used internationally (Luszczynska, Scholz, & Schwarzer, 2005). Internal consistency has been reported to be *α* = .90 (Löve, Moore, & Hensing, 2012), with *α* = .89 in the present study. The silhouette values in the cluster analyses indicated fair cluster solutions and repeated clustering further demonstrated cluster stability. Furthermore, the survey response rate was high and the MI method handling missing data resulted in a maximized sample size. The sample included two universities and one university college which speaks in favour of the generalizability of the results.

## 3 RESULTS

Sample characteristics and study participants’ perceptions of the BSN programme are presented in Table 1. Most (85.2%) of the students in this study were females and their mean age was 27.8 years (*SD* 6.3). Most students (63.4%) did not have previous experience of higher education before entering the nursing programme and slightly more than half of the sample (57.0%) had studied a theoretical programme at upper secondary school while the rest had studied nursing care (15.2%) or another programme (27.9%). Slightly more than half of the students (54.1%) had prior work experience in healthcare. A large proportion (81.1%) worked in healthcare while they were studying. Most students (82.7%) responded that they would likely or definitely recommend their BSN programme to others and most (73.1%) rated the overall quality of their BSN programme as high. On average, students rated their GSE as 3.2 (*SD* = 0.46). About two‐thirds (61.8%) passed the final examination (NCFE). Among the NPC competence areas, Value‐based Nursing Care was rated highest while the competence area Development, Leadership and Organization of Nursing Care was rated lowest (Table 2).

**Table 1:** Clusters in relation to sample characteristics and students’ perceptions of the Bachelor of Science in Nursing (BSN) programme

Variables	Cluster			*p* values
	1/passed *N* = 36	2/passed *N* = 69	3/failed *N* = 65	
Gender, male/female, *N* = 169	6/29	8/61	11/54	.623^b^
Age, years, *N* = 168, mean (*SD*)	29.2 (6.9)	26.9 (5.3)	27.9 (6.9)	.200^a^
General Self‐Efficacy Scale, *N* = 167, mean (*SD*)	2.8 (0.4)	3.3 (0.4)	3.2 (0.5)	<.001^a^
Education at upper secondary school level prior to entering the BSN Programme, *N* = 165, (3‐year theoretical programme Natural Science/3‐year theoretical programme Social Science/3‐year programme Nursing Care/Other programme)	8/14/3/9	9/32/8/19	9/22/14/18	.384^b^
Higher Education before the nursing programme, *N* = 167, yes/no	17/18	22/46	22/42	.243^b^
Work experience in healthcare prior to entering the BSN Programme, *N* = 170, yes/no	19/17	34/35	39/26	.453^b^
Paid work experience in healthcare when studying the BSN Programme, *N* = 169, yes/no	32/4	48/20	57/8	.017^b^
Recommend the BSN programme to others, *N* = 168 (no/likely/definitely)	13/19/4	9/29/30	7/32/25	.001^b^
Overall Quality of the BSN Programme^d^, *N* = 167, Md (Q1;Q3)	2 (2;3)	3 (3;3)	3 (3;3)	<.001^c^

Note

Mean, median (Md), standard deviations (*SD*) and quartiles (Q1, Q3).

a One‐way ANOVA.

b Chi‐square test.

c Kruskal–Wallis test.

d Response alternatives were 1 = very low, 2 = low, 3 = high and 4 = very high.

**Table 2:** Cluster means and standard deviations in relation to NPC competence areas

Variables	Total sample	Cluster 1/passed	Cluster 2/passed	Cluster 3/failed	*P* value^a^
Number of participants (%)	170	36 (21.2%)	69 (40.6%)	65 (38.2%)	
Nursing care	84.9 (11.0)	74.2 (10.4)	89.0 (8.6)	86.5 (9.8)	<.001
Value‐based nursing care	90.6 (10.1)	79.3 (13.0)	95.4 (5.2)	91.8 (7.2)	<.001
Medical and technical care	82.2 (11.1)	70.7 (10.8)	87.8 (7.5)	82.6 (9.7)	<.001
Care pedagogics	81.8 (12.8)	69.4 (11.1)	87.5 (10.6)	82.7 (11.3)	<.001
Documentation and administration of nursing care	83.5 (9.8)	73.5 (8.8)	88.1 (7.0)	84.2 (8.9)	<.001
Development, leadership, and organization of nursing care	71.7 (12.6)	60.6 (8.9)	77.7 (11.3)	71.5 (11.6)	<.001

Note

Values for NPC scores ranged between 1–100, where 100 correspond to high self‐reported competence.

Abbreviation: NPC, Nurse Professional Competence Scale.

a One‐way ANOVA.

**Figure 1:**
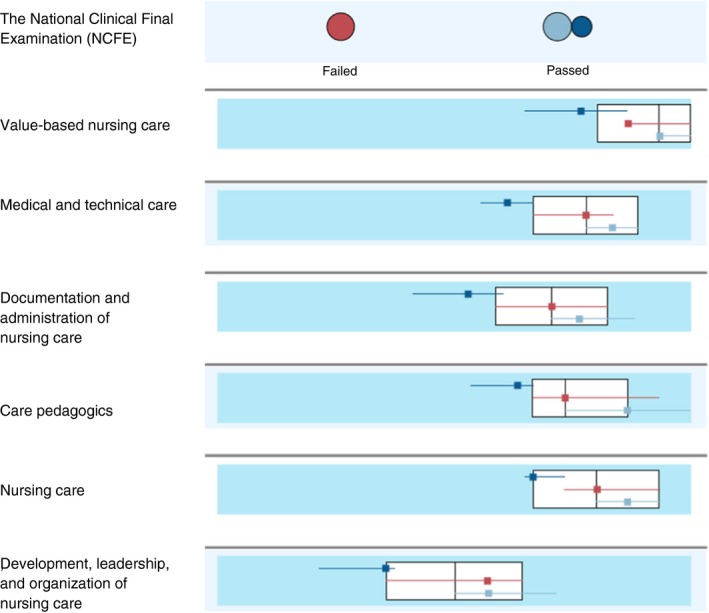
A comparison of the three clusters. Continuous variables (competence areas) are shown as boxplots with the overall medians and interquartile ranges (white) together with boxplots for each cluster’s median and interquartile range. The categorical variable (NCFE) is shown as dot plots (the size indicates the most frequent response for each cluster). Cluster 1 (passed NCFE, dark blue), Cluster 2 (passed NCFE, light blue), and Cluster 3 (failed NCFE, red)

**Figure 2:**
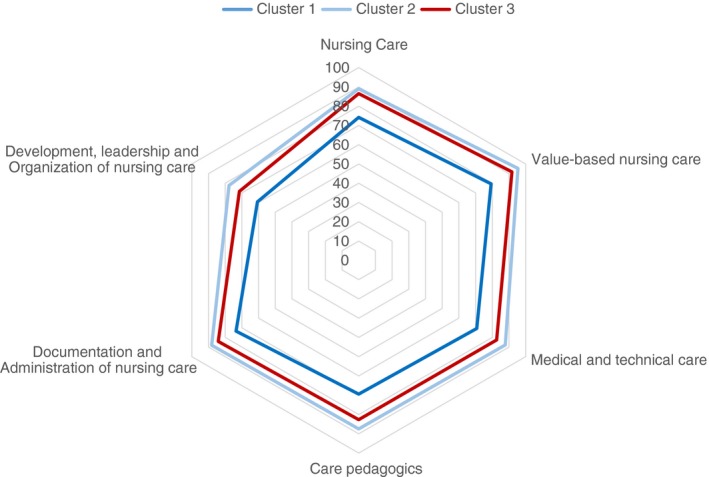
Cluster mean values for NPC competence areas. Cluster 1 (passed NCFE, dark blue), Cluster 2 (passed NCFE, light blue), and Cluster 3 (failed NCFE, red). NPC, Nurse Professional Competence Scale; NCFE, National Clinical Final Examination

A TSCA was performed separately on each of the five MI datasets and showed stability across the different versions. All MI datasets revealed a solution with three clusters with similar patterns. Silhouette measures of cohesion and separation ranged from 0.455–0.463 (indicating a fair solution). The first MI dataset was then kept for further data analyses. The cases were randomly ordered three times and the stability of the cluster solution was verified. For all solutions, there were three clusters with 36, 65, and 69 students and the silhouette measure of cohesion and separation was 0.459 (a fair solution). The three clusters from MI dataset 1 are presented in Table 2 and Figures 1 and 2. The students in cluster 1 (*N* = 36) had all passed the national clinical examination test but rated their competence as significantly lower than students in the two other clusters (Post Hoc, Bonferroni adjusted, all *p* values <.001). Their ratings were below the median for all competence areas (Figure 1; mean values for the competence areas ranged from 60.6–79.3, Table 2). The students in cluster 2 (*N* = 69) had also passed the national clinical examination test. They rated themselves as quite good in all competence areas and above the median for the total sample for five of the six competence areas (mean values ranged from 77.7–95.4). The students in cluster 3 (*N* = 65) had all failed the national clinical examination test. They rated themselves lower than the median for one competence area, higher for one and on the median for the remaining four competence areas (mean values ranged from 71.5–91.8).

Sample characteristics for the clusters and students’ perceptions of the overall quality of their BSN programme are presented in Table 1. Cluster 1 scored significantly lower than clusters 2 and 3 regarding GSE (Post Hoc, Bonferroni adjusted, all *p* values <.001), they rated the overall quality of the BSN programme lower than clusters 2 and 3 (Mann–Whitney *U* test all *p* < .001) and fewer in cluster 1 would definitely recommend the BSN programme (adjusted residual 3.4). Regarding paid work in healthcare while studying the BSN Programme, the results showed that fewer worked in cluster 2 than in the other two clusters (adjusted residual 2.9).

## 4 DISCUSSION

The main result from this study was the identification of three different clusters, which illustrated students’ different competence profiles. Students in cluster 1 passed the NCFE but presented lower self‐assessed competence (NPC) than the overall median values in the group. Students in cluster 2 also passed the NCFE and rated themselves as above median in all but one NPC competence area. Students in cluster 3 failed the NCFE but still rated themselves on the median level or higher in all but one of the NPC competence areas. Thus, self‐assessed and non‐self‐assessed competency were concordant among students in cluster 2, whereas students in clusters 1 and 3 differed in these two aspects in different ways. This disparity might have consequences for these students while in nursing education and also for health care organizations when the students enter working life.

Self‐assessment is a critical skill and also complex and challenging for students. In the literature, this has been studied and discussed in relation to the ‘impostor syndrome’ for example (characterized by chronic degrading feelings) (Villwock, Sobin, Koester, & Harris, 2016), or the opposite, which results in inflated self‐assessments (Kruger & Dunning, 1999). This illustrates how people might exhibit different approaches to self‐assessments of their abilities. In our sample, the students in cluster 1 might be the ones suffering from the ‘impostor syndrome’, that is, their self‐criticism overrides their actual abilities. Their GSE was lower than for students in the other two clusters and they were also more critical to the quality of their education. They performed ‘well enough’ but were still dissatisfied. However, the students in cluster 3 seemed to exhibit inflated self‐assessments in relation to their NCFE results.

Based on these results, we need to consider what pedagogical methods we use in education and how they can strengthen students’ ability to develop their self‐assessment competence. We must also consider how we can educate students based on their specific needs. Educational interventions focusing on self‐directed and self‐regulated learning (Saks & Leijen, 2014) might support students’ ability to self‐assess and self‐reflect, thereby improving their learning conditions. For example, the pedagogical model peer learning, which is characterized by a two‐way, reciprocal learning activity supporting abilities like self‐assessment and peer assessment as well as communication and articulation of own knowledge, understanding, and skills (Boud, Cohen, & Sampson, 2001; Pålsson, Engström, Leo Swenne, & Mårtensson, 2018). In relation to the clinical education of undergraduate nursing and other health science students, peer learning has demonstrated improved nursing self‐efficacy (Pålsson, Mårtensson, Swenne, Adel, & Engström, 2017) and self‐evaluation (Secomb, 2008), for example. Another promising learning intervention is drama. Drama pedagogy has been used in nursing education to prepare students on both bachelor and master level for their future nursing roles (Arveklev, 2017). The use of drama in nursing education can provide opportunities to explore interactions with others, which can increase students’ self‐awareness and their ability to reflect on their future professional identity as nurses. Furthermore, drama allows the students to re‐enact situations, ethical dilemmas, and conflicts from the healthcare context to practice, reflect, discuss, and learn about conflict management (Arveklev, Berg, Wigert, Morrison‐Helme, & Lepp, 2018).

In previous studies on nurses and competence, factors such as length of work experience, frequent use of competence, factors related to the practice environment, and healthcare context, as well as nurse related and sociodemographic factors have been found to be related to the different areas of competence (Flinkman et al., 2017; Gardulf et al., 2016; Leksell, Gardulf, Nilsson, & Lepp, 2015; Meretoja, Numminen, Isoaho, & Leino‐Kilpi, 2015; Nilsson et al., 2016; Salonen, Kaunonen, Meretoja, & Tarkka, 2007). Furthermore, for nursing students, work in healthcare while studying has been found as related to self‐reported competence (Gardulf et al., 2016). Competence is developing over time and depends on work experience, which is also formulated by Benner (1989) who describes the process from novice to expert. The newly graduated nurse is a beginner, which is important to consider in healthcare organizations and for the newly graduated themselves. Results from this study illustrate how students’ self‐assessments and beliefs in their competence might differ from actual performance. This is also important to consider when the new nurse enters the healthcare organization. Correct assessments of one's own competence/ability are essential for patient safety.

The understanding of lifelong learning in relation to nursing research and curricula is crucial to support the necessary skills and attitudes among the students (Davis, Taylor, & Reyes, 2014). Qalehsari, Khaghanizadeh, and Ebadi (2017) have highlighted the complexity of lifelong learning and concluded that one single strategy cannot lead to lifelong learning alone. The use of strategies for lifelong learning will lead to increased quality of education and of patient care and to the development of nursing competency.

### 4.1 Limitations

Analyses were based on overall competency rather than on specific areas of competence, which can be considered as a limitation and this needs further study. However, the cluster approach illustrating different competence profiles for subgroups of students has its advantages. Weaknesses with the TSCA are that the final solution might depend on the order of cases and thereby the method should be tested with the cases in different order (Norusis, 2012). The TSCA was therefore applied three times with the cases in different random order to test the stability of the cluster solution. Generalizability of the results must be done with caution as the sample included only three out of 25 HEI in Sweden.

## 5 CONCLUSION

This study illustrates how nursing students’ self‐assessed competence might differ from competency assessed by examination. This is challenging for nursing education, both for theoretical parts of the nursing programme and for clinical education. Pedagogical interventions to support realistic perceptions of own competence are crucial since the perceptions that students and later on registered nurses, have of their own competence might be a matter of patient safety.

## CONFLICT OF INTEREST

No conflict of interest has been declared by the authors.

## AUTHOR CONTRIBUTIONS

All authors have made substantial contributions to all of the following: (1) the conception and design of the study, or acquisition of data, or analysis and interpretation of data, (2) drafting the article or revising it critically for important intellectual content, and (3) final approval of the version to be submitted.
